# Association between Change in Regional Phase Angle and Jump Performance: A Pilot Study in Serie A Soccer Players

**DOI:** 10.3390/ejihpe11030063

**Published:** 2021-08-15

**Authors:** Tindaro Bongiovanni, Athos Trecroci, Alessio Rossi, Fedon Marcello Iaia, Giulio Pasta, Francesco Campa

**Affiliations:** 1 Department of Health, Nutrition and Exercise Physiology, Parma Calcio, 1913 Parma, Italy; 2 Department of Biomedical Sciences for Health, Università degli Studi di Milano, 20129 Milano, Italy; athos.trecroci@unimi.it (A.T.); marcello.iaia@unimi.it (F.M.I.); 3 Department of Computer Science, University of Pisa, 56126 Pisa, Italy; alessio.rossi@di.unipi.it; 4 Medical Department, Parma Calcio, 1913 Parma, Italy; ghitopasta@hotmail.com; 5 Department for Life Quality Studies, Università degli Studi di Bologna, 47921 Rimini, Italy; francesco.campa3@unibo.it

**Keywords:** body composition, bioelectrical impedance analysis, BIA, football

## Abstract

*Purpose:* This observational longitudinal investigation aimed to investigate whether change in bioelectrical regional phase angle (PhA) is a predictor of change in vertical jump performance in elite soccer players. *Methods*: Fifteen soccer players (age: 28.7 ± 5.0 years, body weight: 82.4 ± 6.8 kg, height: 186.0 ± 0.1 cm, body mass index: 23.8 ± 1.2 kg/m^2^) competing in the first Italian division (Serie A) were included in this study and tested before the pre-season period and after the first half of the championship. Whole body and lower hemisoma PhA were obtained with a phase-sensitive 50 kHz bioelectrical impedance analyzer and legs lean soft tissue was estimated using specific bioimpedance-based equation developed for athletes. Vertical jump performance was assessed using the countermovement jump (CMJ). Results: The major findings of the study are that changes in lower hemisoma PhA are more strongly related with changes in jump performance (r^2^ = 0.617, *p* = 0.001) than changes in whole-body PhA (r^2^ = 0.270, *p* = 0.047), even after adjusting for legs lean soft tissue and for body mass index (β = 5.17, *p* = 0.004). *Conclusions:* These data suggest that changes in lower hemisoma PhA might be used as a tool for evaluating performance related parameters in sports where specific body segments are involved, in preference to the whole-body measured value.

## 1. Introduction

In elite soccer players, higher levels of strength and power are now required in order to reproduce intense muscular bursts such as accelerations and decelerations, maximal sprinting (30–40 times), turning (>700 times), tackling and jumping (30–40 times) [1]. Notably, vertical jump performance is utilized to assess isometric and dynamic strength and is widely used in prescribing lower limb explosive strength training in elite soccer players [2].

It is well known how different physical performance parameters including jumps are related to body composition features [3,4]. Interestingly, the ratio between intracellular and extracellular water, as well as cellular integrity and density have recently been shown to be associated with soccer-related performance in elite players [5,6]. In particular, body fluid distributions and cellular health are reflected in the bioelectric phase angle (PhA), an easy to obtain bioelectrical value [7]. It has been shown that PhA is higher in soccer players than general population, while differences between athletes and controls seem to vary according to the sport/physical activity modality [7]. In this regard, physical activity has been shown to have a positive effect on PhA, which is higher in active individuals and also significantly increases in active subjects in longitudinal studies when compared with controls [3,7].

Recently, Nabuco et al. [6] reported the relationship between whole-body PhA and sprint performance, also highlighting how it is inversely correlated with perceived fatigue in soccer players. Compared with conventional, whole-body PhA, the regional PhA allows one to evaluate the composition of specific body segments, overcoming the theoretical limits underlying conventional analyses such as the idea of the human body as the set of five cylinders of uniform resistivity [7]. Recent studies suggest that regional PhA can provide important information about changes in body fluids among the different hemisomes, which can occur after a muscle injury [8], or as a result of an increase in the number and/or size of cells achieved after a training program [9,10]. However, the use of regional PhA as an indicator of the performance of athletes from sports that predominantly involve specific body segments has yet to be determined.

From a practical perspective, PhA represents a simple, quick, and non-invasive value that could be used to monitor the overall fitness and body composition features of the players during the competitive season. The assessment of PhA can be considered together with other parameters related to physical performance, such as muscle strength and endurance level, in the search for the player’s optimal physical condition. In addition, since PhA can be measured either whole-body or in the different hemisomes, it is important to study which measure may be more informative in the evaluation of a soccer player. Therefore, the aim of this study was to verify, through a longitudinal pilot study design, the association between changes in lower hemisoma PhA and vertical jump in elite soccer players. Our hypothesis was that change in lower hemisoma PhA could be more strongly related with change in vertical jump performance than that concerning the whole body PhA.

## 2. Materials and Methods

### 2.1. Participants

Seventeen male professional soccer players of the same team attending the Italian first division (Serie A) were selected to participate in this study. Two subjects were excluded as they received injuries before starting the experimental protocol. Therefore, fifteen players (age: 28.7 ± 5.0 years, body weight: 82.4 ± 6.8 kg, height: 186.0 ± 0.1 cm, body mass index (BMI): 23.8 ±1.2 kg/m^2^) took part in the study. The players voluntarily decided to participate and provided a signed informed consent after a detailed description of the study procedures. The project was conducted according to the Declaration of Helsinki and was approved by the Bioethics Committee of the University of Milan.

### 2.2. Procedures

Biolectrical whole body and lower hemisoma PhA (LPhA) were obtained using a phase-sensitive segmental bioelectrical analyzer (BIA 101 BIVA PRO, Akern, Florence, Italy) at a single frequency of 50 kHz. After cleaning the skin with isotropy alcohol, four low intrinsic impedance adhesive electrodes (Biatrodes Akern Srl, Florence, Italy) were placed on the right hand and foot respecting the standard protocol [7,11]. PhA values were measured directly and automatically for both whole body and lower hemisoma and the values were given in degree. The device was calibrated every morning using the standard control circuit supplied by the manufacturer that has a known impedance (Rz ¼ 380 Ohm 1% precision, and Xc ¼ 47 Ohm 1% precision). BIA measurements were all taken by the same trained investigator to avoid inter-observer errors, as previously undertaken [12]. Legs lean soft tissue (LLST) was estimated using a specific equation developed for athletes [13], designed and validated with the same BIA model. The participants were instructed to avoid physical activity in the 3 h prior to the tests and to refrain from consuming alcohol and caffeine for at least 24 h [7].

In the 24 h period before executing the countermovement jump (CMJ) test, participants did not engage in activity that was considered unduly fatiguing. Each participant executed three maximal jump trials on a portable force platform (Quattro Jump, Kistler, Winterthur, Switzerland). Each trial was performed from a standing position with hands placed on the hips. During an individual trial, the participants quickly bent downward and then performed a fast upward push to reach the highest height from the lower limbs action. CMJ height, power output, and strength were recorded within the upward phase by a dedicated software (Kistler software, Version 1.1.1.4). A recovery of 2 min was allowed between trials. The best performance was used within the analysis. Body composition and jump assessments were performed in the morning at the same time (9.00 a.m.) before the pre-season period and after the first half of the championship.

### 2.3. Statistical Analysis

Statistical analysis was performed using IBM SPSS Statistics for Mac OS version 22.0, 2010 (SPSS Inc., IBM Company, Chicago, IL, USA). Descriptive statistics (mean ± standard deviation) were performed for all measurements. All variables were checked for normality using the Shapiro–Wilk test and the results were normally distributed. Regression analysis was used to determine whether change in PhA was a significant predictor of change in jumping performance after adjusting for confounding variables (LLST and BMI). Changes in bioelectrical, body composition, and jump parameters were compared with the student’s *t* test for paired data. Effect sizes were calculated as the mean difference standardized by the between-subject standard deviations. Statistical significance was predetermined as *p* < 0.05.

## 3. Results

Bioelectrical LPhA and jump performance significantly (*p* < 0.05) increased from the pre-season phase to after the first half of the competitive period in the participants, whereas no change was assessed for whole body PhA, BMI and LLST, as shown in Table 1.

**Figure 1:**
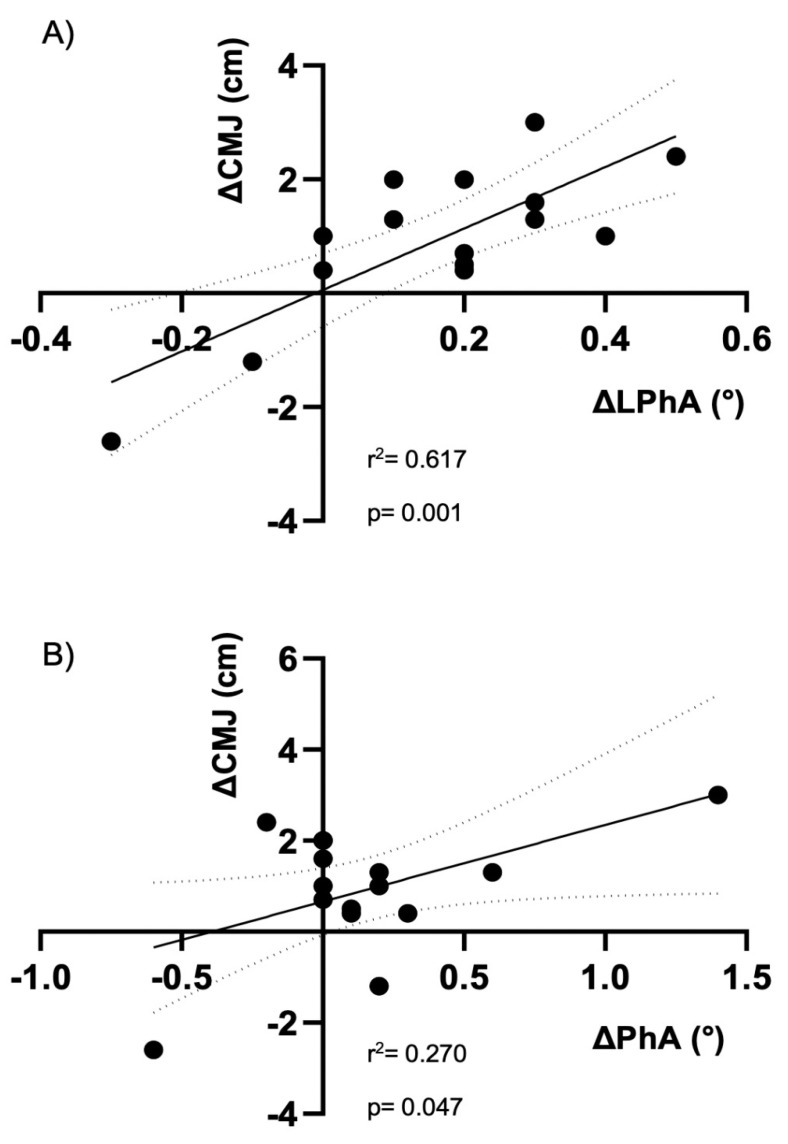
Scatterplots showing the associations between changes (Δ) in lower limb (LPhA) and whole-body phase angle (PhA) and Δ in countermovement jump (CMJ), in panel **A** and **B**, respectively.

Linear regression results showed that change in LPhA was a better predictor of change in CMJ than change in whole body PhA, as shown in Figure 1.

A multiple regression analysis was performed while adjusting the relationship between changes in LPhA and changes in CMJ for confounding variables, as shown in Table 2. Changes in LPhA alone explained ~60% of change in jump performance variance (β = 5.40; *p* = 0.001). After adjusting for LLST and BMI, change in LPhA remains a significant predictor in the model (β = 5.17; *p* = 0.004), as shown in Table 2. 

## 4. Discussion

The aim of this investigation was to test the association between changes in lower hemisoma PhA and vertical jump in elite soccer players. As hypothesized, lower hemisoma PhA is a better predictor of vertical jump performance than whole body PhA. In particular, increases in lower hemisoma PhA are associated with an improvement in CMJ capacity. To the authors’ knowledge, this is the first study to investigate the longitudinal association of regional PhA with performance parameters, regardless of their important body components such as lean soft tissue mass and BMI. 

The result of the regression analysis identified the change in lower heimsoma PhA as a significant predictor of change in vertical jump performance, accounting for ~60% of inter-individual variance. In the study by Nabuco et al. [6] the association between whole-body PhA and physical performance measures lose significance when adjusted for lean body mass. On the contrary, in our study after adjusting for co-variables, such as lean soft tissue and BMI, the explained variability increases by up to 65%. The inclusion of legs lean soft tissue and BMI increases the explained variance, without compromising the predictive power of the lower hemisoma PhA. Higher PhA values reflect cellular integrity and a higher muscle mass [11] and this may be predisposing the athlete to express greater strength in the jump. Although our study, at the best of our knowledge, is the first to consider associations between regional PhA and physical performance, previous studies have already investigated the correlations between whole body PhA and sprint performance in soccer players [5,6]. However, in sports where specific body segments are more involved, such as legs in soccer, a regional assessment of body composition characteristics appears to be more informative than a traditional analysis.

The present investigation has some limitations. In fact, our results cannot be compared with those obtained from bioimpedance measurements performed using different technology and sampling frequency than the ones used here. In addition, future studies including a larger sample size and a wider battery of tests are required to investigate the role and usefulness of regional PhA in predicting soccer-specific performance.

Many studies report the negative influence of sub-par body composition features (e.g., dehydration status, higher fat mass, and lower lean and muscle mass) on physical and mental performance, which not only compromise normal daily activities but can negatively affect sports performance [7]. This study suggests that BIA according to a regional approach can be a useful tool for evaluating performance related parameters in sports where specific body segments are involved, preferring it to the whole-body measured value. In addition, lower limb PhA data could help strength and conditioning coaches in monitoring and prescribing lower limb explosive strength training in elite soccer players. 

## 5. Conclusions

In some sports where particular body segments are predominantly involved, a regional PhA evaluation may be more informative than the same value assessed using a whole-body approach in predicting physical performance. This pilot study, conducted during a competitive Serie A soccer season, suggests that an increase in lower limb PhA appears to be associated with an increase in vertical jump performance in elite soccer players. 

## Figures and Tables

**Table 1:** Bioimpedance, body composition, and jump parameters before (PRE) preparatory period and after (POST) the first half of the season.

Variable	PRE	POST	95% CI	t	*p*-Value	ES
BMI (kg/m^2^)	23.8 ± 1.2	24.0 ± 1.2	−0.39, 0.01	−1.9	0.066	0.49
LLST (kg)	28.2 ± 2.6	28.1 ± 3.1	0.44, 0.75	0.6	0.586	−0.14
PhA (°)	7.9 ± 0.5	8.0 ± 0.4	−0.35, 0.09	−1.2	0.236	0.31
LPhA (°)	8.2 ± 0.5	8.3 ± 0.5	−0.25, −0.03	−2.8	0.014	0.69
CMJ (cm)	49.5 ± 7.8	50.1 ± 4.8	−1.63, −0.20	−2.7	0.016	0.68

Note: Data are presented as mean ± standard deviation. CI: confidence intervals. ES: effect size. BMI: body mass index. LLST: legs lean soft tissue. PhA: whole body phase angle. LPhA: lower hemisoma phase angle. CMJ: countermovement jump.

**Table 2:** Regression analysis between changes (Δ) in independent variables and Δ in jump performance.

Independent Variable	R^2^	SEE	β	95% Confidence Interval	*p*-Value
ΔLPhA	0.617	0.89	5.40	2.85, 0.70	0.001
Model 1	0.639	0.90	5.65	2.98, 8.33	0.001
Model 2	0.657	0.92	5.17	2.08, 8.26	0.004

Note: R^2^: Coefficient of determination. SEE: Standard error of estimation. β: Standardized coefficients beta. LPhA: lower hemisoma phase angle. Model 1: adjusted for legs lean soft tissue. Model 2: adjusted for legs lean soft tissue and body mass index.

## Data Availability

The data that support the findings of this study are available from the corresponding author upon reasonable request.
